# Supplementary material for: Antibiofilm Combinatory Strategy: Moxifloxacin-Loaded Nanosystems and Encapsulated N-Acetyl-L-Cysteine
Source: Pharmaceutics. 2022 Oct 26;14(11):2294. doi: 10.3390/pharmaceutics14112294 (PMC9699636; doi:10.3390/pharmaceutics14112294)
Supplement: Supplementary file 1 [file pharmaceutics-14-02294-s001.zip › pharmaceutics-1969049-supplementary.pdf]

pharmaceutics

Supplementary materials: Antibiofilm Combinatory Strategy: Moxifloxacin-Loaded Nanosystems and Encapsulated N-Acetyl-L-Cysteine

Rita M. Pinto [1,2], Catarina Leal Seabra [1], Martine De Jonge [2], M. Cristina L. Martins [3,4], Patrick Van Dijck [2], Salette Reis [1] and Cláudia Nunes [1,3,*]

[1] LAQV, REQUIMTE, Departamento de Ciências Químicas, Faculdade de Farmácia, Universidade do Porto, 4050-313 Porto, Portugal; anaritapinto5@gmail.com (R.M.P.); cseabra@ff.up.pt (C.L.S.); shreis@ff.up.pt (S.R.)
[2] Laboratory of Molecular Cell Biology, Institute of Botany and Microbiology, KU Leuven, 3001 Leuven, Belgium; martine.dejonge@kuleuven.be (M.D.J.); patrick.vandijck@kuleuven.be (P.V.D.)
[3] Instituto de Ciências Biomédicas Abel Salazar, Universidade do Porto, 4050-313 Porto, Portugal; cmartins@ineb.up.pt
[4] i3S, Instituto de Investigação e Inovação em Saúde INEB, Instituto de Engenharia Biomédica, Universidade do Porto, 4200-135 Porto, Portugal
* Correspondence: cdnunes@ff.up.pt

1. Determination of the MIC and MBC values for MOX-loaded nanosystems

Table S1: Activity of the developed MLNs and free MOX against planktonic *S. aureus* strains. MIC and MBC values are expressed in μg mL^{-1} of MOX.

	MRSA ATCC 33591		ATCC 25923		ATCC 6538		*Xen36*	
	MIC	*MBC*	*MIC*	*MBC*	*MIC*	*MBC*	*MIC*	*MBC*
uMLNs	>2	>2	>2	>2	>2	>2	>2	>2
MOX-MLNs	0.125	0.125	0.06	0.125	0.25	0.25	0.125	0.125
F-uMLNs	>2	>2	>2	>2	>2	>2	>2	>2
F-MOX-MLNs	0.125	0.125	0.06	0.125	0.125	0.125	0.125	0.125
MOX	0.06	0.125	0.03	0.06	0.125	0.125	0.06	0.06

2. In vitro antibiofilm activity: biofilm viability and biofilm biomass study in *S. aureus* ATCC 25923 biofilms

The antibiofilm efficacy of both MOX- and NAC-loaded nanosystems against the *S. aureus* strain ATCC 25923 was assessed by the XTT and crystal violet assays (**Figure S1**). Similar to the results obtained for the other three tested strains, biofilms treated with both unloaded and NAC-loaded LNPs showed a significant reduction on bacterial viability. However, free NAC does not seem to have a significant effect at the concentrations tested. Regarding biofilm biomass, only formulations or free NAC at the highest concentration (2 mg mL^{-1}) showed a significant reduction compared to the untreated control.

The developed MOX-MLNs and F-MOX-LNPs showed a high effect on reducing bacterial viability at drug concentrations as low as 0.125 μg mL^{-1}, which was not verified for the corresponding unloaded formulations. At the lowest concentration tested, free MOX also exhibited a high effect on viability. These results were further confirmed by the crystal violet assay. In this study, encapsulated and free MOX exhibited a biofilm biomass reduction higher than 50% at the lowest concentration tested. For the unloaded vehicles, only at the highest concentrations it is possible to observe a significant difference in the biofilm biomass, compared to the control.

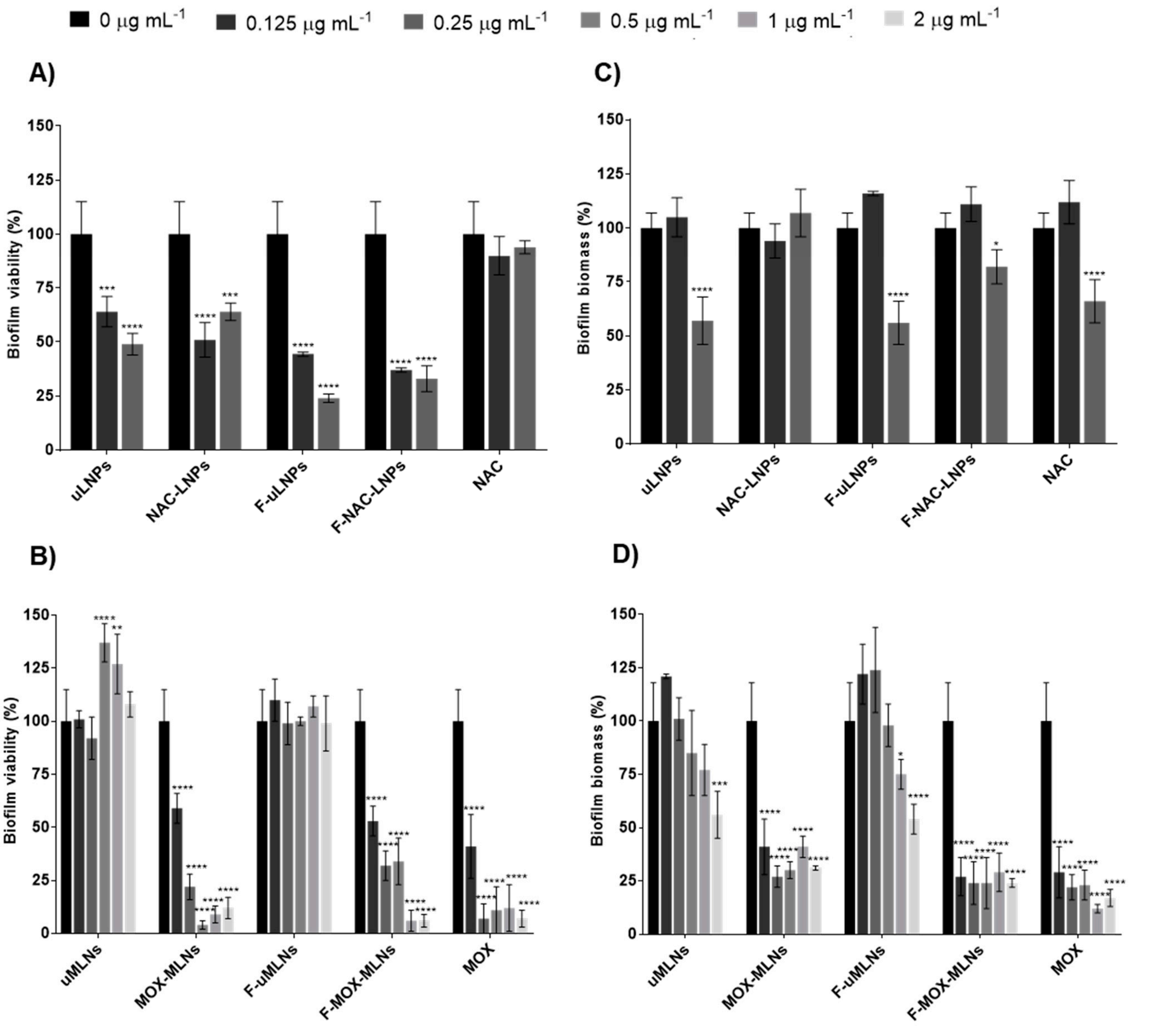

Figure S1: Quantification of **(A, B)** biofilm viability (XTT assay) and **(C, D)** biomass (crystal violet staining) after a 24h treatment of *S. aureus* ATCC 25923 biofilms. **(A, C)** The biofilms were treated with unloaded and NAC-loaded LNPs at the solid lipid concentrations of 0, 1, and 2 mg mL^{-1}, which corresponds to 0, 0.45, and 0.9 mg mL^{-1} of NAC, respectively. Free NAC was tested at the same concentrations. **(B, D)** The biofilms were treated with the developed MLNs and free MOX at the concentrations of 0, 0.125, 0.25, 0.5, 1, and 2 µg mL^{-1} of MOX. Untreated biofilms (0 µg mL^{-1}) were used as a positive control and TSB 0.6x supplemented with 0.2% (w/v) glucose was used as a negative control. The values are represented as the mean ± SD. *$p<0.05$, **$p<0.01$, ***$p<0.001$, ****$p<0.0001$ relatively to 0 µg mL^{-1}. Statistical analysis: two-way ANOVA, Tukey's multiple comparisons test.
